# 离子色谱指纹图谱分析在烟用香精香料品质评价中的应用

**DOI:** 10.3724/SP.J.1123.2023.11008

**Published:** 2024-08-08

**Authors:** Gaoyan XU, Xinying HE, Lina ZHANG, Chongsheng LIU, Yang GAO, Zhongping HUANG, Huijun LIU, Zhaoming WU, Ruichao ZHANG, Hong SHI

**Affiliations:** 1.浙江中烟工业有限责任公司技术中心, 浙江 杭州 310008; 1. Technical Center, China Tobacco Zhejiang Industry Co., Ltd., Hangzhou 310008, China; 2.浙江工业大学化学工程学院, 浙江 杭州 310014; 2. College of Chemical Engineering, Zhejiang University of Technology, Hangzhou 310014, China

**Keywords:** 离子色谱法, 烟用香精香料, 色谱指纹图谱, 聚类分析, 相似度分析, ion chromatography (IC), tobacco flavors, chromatographic fingerprint, hierarchical cluster analysis, similarity analysis

## Abstract

为保证烟用香精香料品质的一致性,采用离子色谱法对烟用香精香料样品中的9种有机酸及7种无机阴离子进行分析。1.0 g样品中加入10 mL去离子水,振荡萃取30 min后,水相溶液用0.45 μm微孔滤膜过滤,滤液进一步采用RP前处理柱除去基质,经离子色谱仪分离测定。采用Dionex IonPac AS11-HC阴离子交换柱及淋洗液梯度洗脱,9种有机酸和7种常规无机阴离子标准工作溶液的保留时间和峰面积日内精密度(RSD)分别为0.01%~0.69%和1.34%~2.98%,日间RSD分别为0.03%~0.68%和3.54%~5.16%。同时对烟用香精香料标准样品A~D (各5个批次)中有机酸和无机酸的保留时间和峰面积RSD进行考察,得到的保留时间和峰面积日内RSD分别为0.01%~0.71%和2.39%~3.22%,日间RSD分别0.05%~0.81%和3.61%~6.02%。建立了4种烟用香精香料(各5个批次)的指纹谱图库,并采用系统聚类分析和相似度分析法对其他生产厂家样品进行品质评价。结果表明,聚类分析法能有效地从不同生产厂家的产品中筛选出与标准样品质量最相近的样品;实际样品AY3、BY2、CY2及DY1与香精香料标准品性质最接近,品质较好。相似度分析可以对不同生产厂家的产品质量进行更加具体、量化的评价,实际样品AY3、BY2、CY2及DY1与香精香料标准品的化学成分相似度值均大于97.7%,符合相似度评价要求。相较于超声辅助液液萃取-气相色谱法,对于个别特殊样品,离子色谱法能更有效地区分香精香料样品间的品质差异。

## Abstract (en)

Tobacco flavor, an important tobacco additive, is an essential raw material in cigarette production that can effectively improve the quality of tobacco products, add aroma and taste, and increase the suction flavor. The quality consistency of tobacco flavors affects the quality stability of branded cigarettes. Therefore, the quality control of tobacco flavors is a major concern for cigarette and flavor manufacturers. Physical and chemical indices, odor similarity, and sensory efficacy are employed to evaluate the quality of tobacco flavors, and the analysis of chemical components in tobacco flavors is usually conducted using gas chromatography (GC) and high performance liquid chromatography (HPLC). However, because the composition of tobacco flavors is complex, their quality cannot be fully reflected using a single component or combination of components. Therefore, establishing an objective analytical method for the quality control of tobacco flavors is of extreme importance. Chromatographic fingerprint analysis is routinely used for the discriminative analysis of tobacco flavors. Chromatographic fingerprints refer to the general characteristics of the concentration profiles of different chemical compounds. In the daily procurement process, fingerprints established by GC and HPLC are effective for the evaluation and identification of tobacco flavors. However, given continuous improvements in aroma-imitation technology, some flavors with high similarity cannot be directly distinguished using existing methods.

In this study, a method for the determination of organic acids and inorganic anions in tobacco flavors based on ion chromatography (IC) was developed to ensure the quality consistency of tobacco flavors. A 1.0 g sample of tobacco flavors and 10 mL of deionized water were mixed and vibrated for 30 min. The aqueous sample solution was passed through a 0.45 μm membrane filter and RP pretreatment column in succession to eliminate interferences and then subjected to IC. Standard solutions containing nine organic acids and seven inorganic anions were used to identify the anions in the tobacco flavors, and satisfactory reproducibility was obtained. The relative standard deviations (RSDs) for retention times and peak areas were <0.71% and <6.02%, respectively. The chromatographic fingerprints of four types of tobacco flavors (samples A-D) from five different batches were obtained. Nine tobacco flavor samples from different manufacturers (samples AY1-AY3, BY1-BY2, CY1-CY2, DY1-DY2) were also analyzed to obtain their chromatographic fingerprints. Hierarchical cluster and similarity analyses were used to evaluate the quality of tobacco flavors from different manufacturers. Hierarchical clustering refers to the process of subdividing a group of samples into clusters that exhibit a high degree of intracluster similarity and intercluster dissimilarity. The dendrograms obtained using SPSS 12.0 indicated good quality consistency among the samples in different batches. Samples AY3, BY2, CY2, and DY1 clustered with the batches of standard tobacco flavors. Therefore, hierarchical cluster analysis can effectively distinguish the quality of products from different manufacturers. The Similarity Evaluation System for Chromatographic Fingerprint of Traditional Chinese Medicine (version 2.0) was used to evaluate the similarity between the standard tobacco flavors and products from different manufacturers. Among the samples analyzed, samples AY3, BY2, CY2, and DY1 showed the highest similarity values (>97.7%), which was consistent with the results of the hierarchical cluster analysis. This finding indicates that IC combined with chromatographic fingerprint analysis could accurately determine the quality of tobacco flavors. GC combined with ultrasonic-assisted liquid-liquid extraction was also used to analyze the tobacco flavors and verify the accuracy of the proposed method. Compared with GC coupled with ultrasonic-assisted liquid-liquid extraction, IC demonstrated more significant quality differences among certain tobacco flavors.

烟用香精香料作为重要的烟草添加剂之一,是卷烟生产中必不可少的原料,其能有效改善烟草制品的品质,添香矫味,增加抽吸香味^[1,2]^。因此烟用香精香料的品质一致性将影响品牌香烟的质量稳定性,香精香料生产企业及采购商都需严格监控其质量。目前,在烟用香精香料品质鉴定过程中,通常对物化指标和嗅香相似度、感官功效作用进行评价分析,并结合气相色谱法(gas chromatography, GC)和高效液相色谱法(high performance liquid chromatography, HPLC)分离检测样品中的化学成分^[3,4]^。由于香精香料的成分复杂,样品中的单一组分或者少量组分已不能全面反应样品的品质,因而有研究报道采用色谱指纹图谱分析法,同时从宏观整体性和指纹特征性考察烟用香精香料样品间的异同,对样品中的复杂组分进行全面比较^[5⇓-7]^。

目前烟草公司采用GC、HPLC等方法建立的指纹图谱对烟用香精香料质量的评估鉴定工作卓有成效,然而随着仿香技术的不断提升,在最近几轮采购工作开展过程中,也发现存在一些相似度较高的香精香料用现有方法难以直接分辨的情况。GC对香精香料中挥发性组分(如萜、醇、酯、烃等)的分析发挥出了极大的优势,但却不适用于非挥发性组分的检测,如溶剂萃取相中的有机酸以及浸膏样品的固态、半固态组分^[8⇓-10]^。HPLC可用于不挥发及热不稳定性物质的分离,而紫外检测器仅对部分物质有较高的灵敏度,如含共轭结构的物质,但对有机酸及无机阴离子的响应较低^[11,12]^。本研究拟从有机酸及无机阴离子角度开展成分研究,解决个别香精香料样品难以准确评判其品质的问题,在原有鉴定方法基础上补充离子色谱法(ion chromatography, IC),开发一套更加全面、有效的化学成分分析方法,完善烟用香精香料指纹图谱评价体系,为后续香精香料招投标工作及日常遇到的个别问题样品的品质鉴定提供技术保障。

针对复杂基质样品中有机酸和无机阴离子的分析,通常采用GC和IC。在采用GC分析有机酸时,需要对目标物进行衍生化前处理,操作过程比较复杂^[13,14]^; IC适用于有机酸和无机阴离子的同时分析,并且前处理简单、无需有机试剂洗脱等,已成为分析工作者检测阴离子的首选方法^[15⇓⇓-18]^。已有文献报道采用IC分析烟用香精香料中有机酸及无机阴离子^[19]^,但尚未有基于烟用香精香料中有机酸及无机阴离子进行色谱指纹图谱研究并应用于香精香料产品质量监控的报道。本文采用IC建立了烟用香精香料中9种有机酸和7种常规无机阴离子的分离方法,获得指纹图谱数据并对所得数据进行统计分析。应用软件SPSS.12及中药色谱指纹图谱相似度评价系统2.0对4种香精香料不同批次样品和不同生产厂家样品进行聚类分析和相似度评价,拟以烟用香精香料中有机酸、无机酸为指标,建立评价烟用香精香料样品质量的有效方法,完善原有采用气相色谱法及液相色谱法建立的烟用香精香料品质评价体系。

## 1 实验部分

### 1.1 仪器与试剂

ICS600离子色谱仪(美国赛默飞世尔科技有限公司),配备RFC-30淋洗液发生器、电导检测器、AERS-500阴离子再生抑制器;Milli-Q Element A10超纯水机(美国Millipore公司);振荡器(常州诺基仪器有限公司); RP型固相萃取小柱(赛默飞世尔科技(中国)有限公司);有机相针式滤器(尼龙,13 mm×0.22 μm,上海安谱实验科技股份有限公司);水系微孔滤膜(50 mm×0.45 μm,上海兴亚净化材料厂); SK2510HP超声波振荡仪(中国无锡同建科技有限公司); BP211D电子分析天平(德国Sartorius公司);离心机(中国湖南湘仪集团)。

有机酸离子的标准溶液分别采用优级纯的乳酸、乙酸、丙酸、甲酸、苹果酸、酒石酸、丙二酸、富马酸、柠檬酸配成1000 mg/L的标准储备液A;无机酸离子的标准溶液分别采用NaF、NaCl、NaBr、Na_2_SO_4_、Na_3_PO_4_、NaNO_2_、NaNO_3_配成1000 mg/L的标准储备液B,于4 ℃冰箱中保存。各取1 mL标准储备液A和储备液B于100 mL容量瓶中,混合后用去离子水稀释成10 mg/L的混合标准工作溶液。二氯甲烷、无水乙醇(AR,国药集团化学试剂有限公司);甲醇(AR,上海凌峰化学试剂有限公司); NaCl (AR,上海市四赫维化工有限公司)。实验用水为去离子水(电阻率为18. 2 MΩ·cm)。

实际生产过程中使用的4种烟用香精香料标准样品(A、B、C、D,各5个批次)均由浙江中烟工业有限责任公司提供,并用于建立指纹谱图库。另有不同生产厂家提供的分别与4种烟用香精香料标准样品性质相似的样品,其中样品A有3个不同生产厂家(AY1、AY2、AY3),样品B、C、D都各有2个不同生产厂家(BY1、BY2、CY1、CY2、DY1、DY2),用于品质评价。

### 1.2 样品前处理

在250 mL磨口三角瓶中,准确加入1.0 g香精香料样品(精确至0.0001 g)和10.0 mL去离子水,盖上瓶塞,振荡萃取30 min。水相溶液用0.45 μm微孔滤膜过滤,收集续滤液。移取1.0 mL滤液置于10 mL容量瓶中,用去离子水稀释定容至刻度摇匀,溶液先经0.22 μm有机相针式滤器过滤并通过RP型固相萃取小柱后进行IC分析。

### 1.3 离子色谱条件

色谱柱:Dionex IonPac AS11-HC阴离子交换柱(250 mm×4 mm)、Dionex IonPac AG11-HC保护柱(50 mm×4 mm);淋洗液:KOH梯度淋洗;流速:1.0 mL/min;抑制器电流:112 mA;柱温:30 ℃;电导检测器温度:35 ℃;进样量:10 μL。梯度条件:0~7 min, 2 mmol/L KOH; 7~35 min, 2~45 mmol/L KOH; 35~38 min, 2 mmol/L KOH。

## 2 结果与讨论

### 2.1 色谱条件的选择

有机酸的分离一般采用Dionex IonPac AS11-HC阴离子交换柱,该柱的特点是具有较大的离子交换容量且适宜用氢氧化钠或氢氧化钾作为淋洗液。采用等度淋洗时,由于分离组分较多,各物质保留能力差异较大,易出现多种物质同时洗脱或部分物质保留时间过长等问题。因此本研究采用梯度洗脱分离9种有机酸和7种常规无机阴离子。先采用初始浓度为2 mmol/L的KOH溶液分离弱保留的有机酸和氟离子,随后考察了KOH溶液的浓度对其他离子分离效果的影响。结果发现:在0~7 min时选用2 mmol/L KOH溶液, 7~35 min KOH溶液浓度采用一阶线性梯度增至45 mmol/L, 35~38 min KOH溶液浓度降为2 mmol/L,各有机酸和无机阴离子的峰形尖锐、对称,大部分物质实现基线分离,标准工作溶液的色谱图见图1,表明该方法适用于烟用香精香料样品中有机酸和无机阴离子的分离检测。

**图1:**
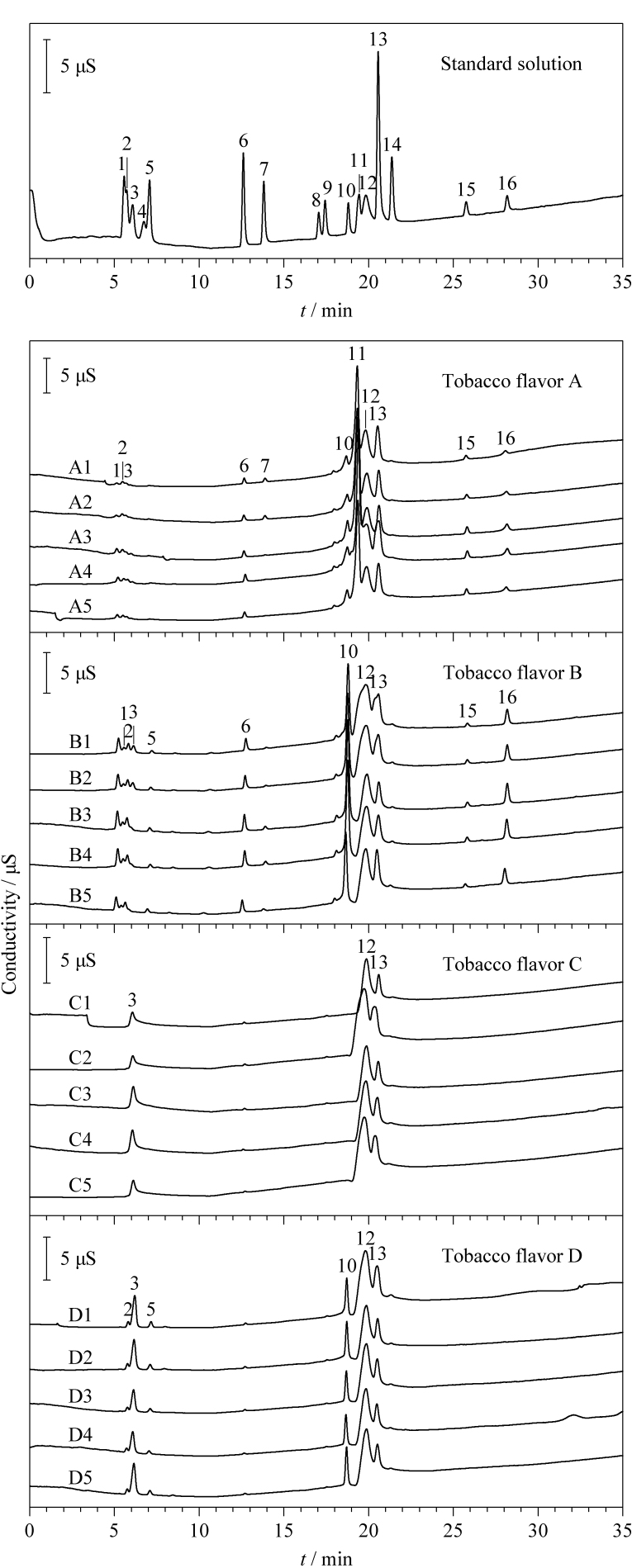
标准混合溶液的色谱图及4种香精香料的色谱指纹图谱

### 2.2 方法学考察

对10 mg/L的混合标准工作溶液进行离子色谱分析并得到各标准物质的保留时间和峰面积。混合标准工作溶液每日平行测定5次,连续测定3日,根据测定结果计算其保留时间和峰面积的相对标准偏差(RSD)。结果表明,9种有机酸和7种常规无机阴离子的保留时间和峰面积日内精密度(RSD)分别为0.01%~0.69%和1.34%~2.98%,日间精密度(RSD)分别为0.03%~0.68%和3.54%~5.16%。同时对样品A~D (各5个批次)中有机酸和无机酸的保留时间和峰面积RSD进行考察,得到的保留时间和峰面积日内RSD分别为0.01%~0.71%和2.39%~3.22%,日间RSD分别0.05%~0.81%和3.61%~6.02%。表明该方法的重复性较好,可用于色谱指纹图谱库的建立。

### 2.3 香精香料标准样品指纹图谱库的建立

根据保留时间对烟用香精香料样品中的各离子进行定性分析。4种烟用香精香料标准样品A~D (各5个批次)的指纹图谱如图1所示。由于不同批次烟用香精香料标准样品中的有机酸和无机阴离子具有很好的重复性,所有5个批次样品色谱图中出现的色谱峰均可作为“共有峰”,因此各色谱峰的保留时间及峰面积作为指纹图谱数据。由烟用香精香料标准样品的色谱图可知,在各香精香料样品中均能检测出多种有机酸和无机阴离子,但其含量存在一定的差异。如样品A与B,存在微量的乳酸、乙酸、F^-^、Cl^-^、 $NO_{2}^{-}$ 、 $NO_{3}^{-}$ 、苹果酸、 $PO_{4}^{3-}$ 、柠檬酸和较高含量的酒石酸、丙二酸、 $SO_{4}^{3-}$ ,然而样品C与D中并未检测到Cl^-^、 $NO_{2}^{-}$ 、 $PO_{4}^{3-}$ 以及柠檬酸。因此,4种烟用香精香料标准样品的指纹图谱具有明显的特征性。

### 2.4 烟用香精香料的聚类分析法评价

采用建立的方法对其他厂家提供的香精香料样品进行分析,根据离子色谱图中的保留时间和峰面积获得指纹图谱数据。将4种烟用香精香料不同批次标准样品及其对应的其他厂家样品的指纹图谱数据分别录入SPSS.12统计软件,采用聚类分析法对其进行数据分析。在方法上采用夹角余弦测量,每两个样本间用组间平均链接法,分别得到了4种样品的相似度矩阵和系统聚类分析图(如图2),这直观地显示了样品逐步合并的过程。由相似度矩阵数据得到,香精香料A~D的各5个批次样品的相关系数分别为0.952~0.997、0.952~0.994、0.938~0.991、0.987~0.998,说明同一样品不同批次之间样品质量稳定性较高,质量波动较小。系统聚类分析图横坐标为临界值即为组间的距离,纵坐标为样品号,临界值越小,表示谱图越相似。以A类型香精香料样品为例,当临界值约为1时,5个不同批次的标准样品A1~A5和AY3聚为一类;当临界值约为5时,AY1与AY2聚为一类。结果表明,AY3样品与A样品属于同一类,质量合格,AY1和AY2不合格。类似的结果,BY2、CY2及DY1与香精香料标准品性质更接近,品质较好。因此,聚类分析法能有效地从不同生产厂家的产品中筛选出与标准样品质量最相近的样品。

**图2:**
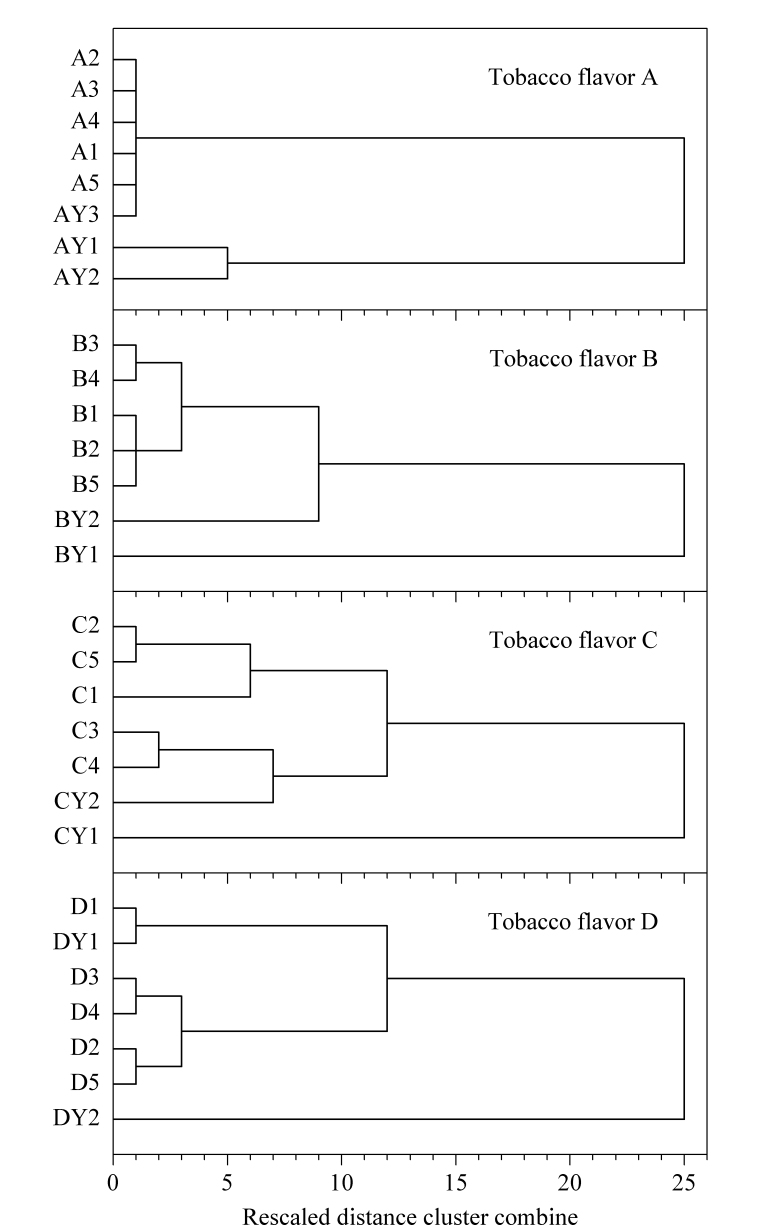
烟用香精香料的系统聚类树系图

### 2.5 烟用香精香料的相似度分析

将4种香精香料标准样品和不同生产厂家样品指纹图谱数据录入到中药色谱指纹图谱相似度评价系统2.0中,进行相似度计算,相关相似度数据结果如表1所示。以A类型香精香料样品为例,AY3和A之间的相似度达到97.9%, AY1、AY2与样品A的相似度为43.7%、39.1%,说明AY3与样品A之间极为相似,AY1和AY2不合格,与2.4节聚类分析结果一致。对于其他3种香精香料样品,BY2、CY2及DY1样品与其对应的标准品间的相似度均大于98%,而BY1、CY1及DY2样品与标准品间的相似度小于92%,该结果也与聚类分析结果一致。依据公司原有的采用气相色谱法及液相色谱法建立的烟用香精香料指纹图谱评价体系,化学成分相似度值≥95%以上的样品进入后续评审。因此,相似度分析可以用于对不同生产厂家的产品质量进行更加具体、量化的评价。

**表1:** 不同生产厂家的烟用香精香料样品的相似度分析

Sample name	Similarity with the tobacco flavor standards/%
AY1	43.7
AY2	39.1
AY3	97.9
BY1	76.6
BY2	98.8
CY1	91.9
CY2	99.9
DY1	99.1
DY2	89.9

### 2.6 液液萃取-气相色谱法与离子色谱法的对比

液液萃取-气相色谱法是分析烟用香精香料样品中挥发性组分最常见和经典的方法。如图3所示,以B类型香精香料样品为例,分别采用杨君等^[7]^研究报道的超声辅助液液萃取-气相色谱法与离子色谱法对标准样品B1及不同生产厂家的BY1和BY2样品进行分析。可以看出,采用超声辅助液液萃取-气相色谱法所得的组分数目明显比采用离子色谱法所得的要少,且液液萃取-气相色谱法所得BY1和BY2与B1的相似度分别为98.7%、100%,样品间的差异较小;离子色谱法所得BY1和BY2与B1的相似度分别为76.6%、98.8%,能更有效区分该类型香精香料样品间的品质差异。

**图3:**
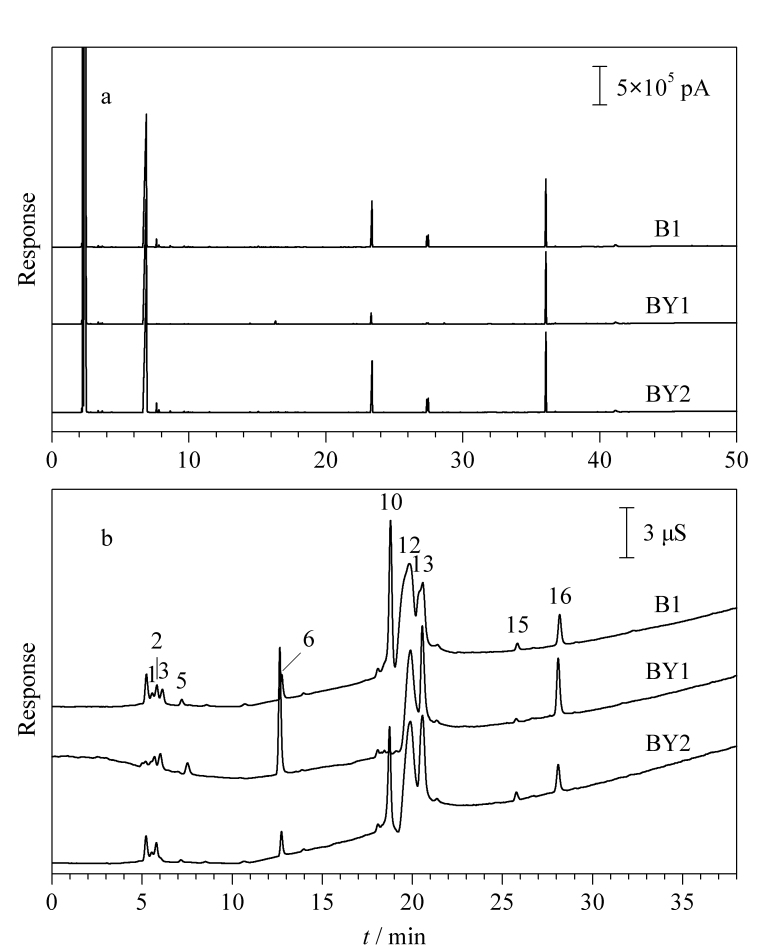
B类型烟用香精香料样品的(a)气相色谱图和(b)离子色谱图

## 3 结论

建立了离子色谱法同时分析烟用香精香料中的9种有机酸和7种无机阴离子的方法。该方法操作简单,重复性好,灵敏度高,适于批量样品分析。将建立的方法应用于测定生产中使用的4种烟用香精香料样品及不同生产厂家样品中的有机酸和无机阴离子,并建立其指纹图谱。结合聚类分析及相似度评价,对4种香精香料不同批次样品和不同生产厂家样品进行品质鉴定。结果表明,离子色谱法结合指纹图谱分析能够准确地鉴别不同香精香料样品的质量。以烟用香精香料中主要有机酸和无机阴离子的分布特征为指标,对于烟用香精香料的质量控制具有重要的作用。

## References

[1] Zhou Z, Shen Y M, Sun J H, et al. China Economist, 2022(2): 293

[2] Cai L L, Zhao Z W, Xi G L, et al. Tobacco Science & Technology, 2020, 53(11): 36

[3] Cai J Y, Li W X, Gu L L, et al. Modern Chemical Industry, 2023, 43(S1): 66

[4] Yu J J, Ding L, Wang Y, et al. Tobacco Science & Technology, 2022, 55(3): 59

[5] Wu W T, Zhu M W, Hu C, et al. Flavour Fragrance Cosmetics, 2023(3): 73

[6] Zhang F Q, Liu Q L, Dong Q J, et al. Guangdong Chemical Industry, 2018, 45(12): 119

[7] Yang J, Jiang J, Xu S Q, et al. Physical Testing and Chemical Analysis Part B: Chemical Analysis, 2012, 48(6): 712

[8] Mayira A, Zhong Z H, Bai X. Chinese Journal of Chromatography, 2023, 41(1): 37 36633075 10.3724/SP.J.1123.2022.04013 PMC9837673

[9] Xu D M, Wu Y F, Wang Y F, et al. Chinese Journal of Chromatography, 2023, 41(1): 76 36633079 10.3724/SP.J.1123.2022.03043 PMC9841437

[10] Wu Y J, Qu B L, Hou Y L, et al. Chinese Journal of Chromatography, 2022, 40(5): 452 35478004 10.3724/SP.J.1123.2021.10018 PMC9404184

[11] Qian Z M, Wu M Q, Tan G Y, et al. Chinese Journal of Chromatography, 2023, 41(8): 690 37534556 10.3724/SP.J.1123.2023.03018 PMC10398825

[12] Yang Z, Lü J X, Wu Y D, et al. Chinese Journal of Chromatography, 2023, 41(7): 602 37387281 10.3724/SP.J.1123.2022.10014 PMC10311626

[13] Tang X D, Su Y, Zhang L N, et al. Tobacco Science & Technology, 2021, 54(4): 49

[14] Liu R H, Pan L N, Wang X Y, et al. Chemical Analysis and Meterage, 2022, 31(12): 22

[15] Zhu Y, Zhu Y, Wang L L. Chinese Journal of Analysis Laboratory, 2011, 30(12): 81

[16] Kanyanee T, Tianrungarun K, Somboot W, et al. Food Chem, 2022, 382: 132055 35255354 10.1016/j.foodchem.2022.132055

[17] Pang J F, Huang Y M, Liu Y L, et al. J Chromatogr A, 2023, 1706: 464231 37517316 10.1016/j.chroma.2023.464231

[18] Hungerford N L, Yates H S A, Smith T J, et al. J Food Compos Anal, 2023, 122: 105466

[19] Li M L, Xi H, Fu Y J, et al. Tobacco Science & Technology, 2022, 55(6): 42

